# Supplementary material for: Personal values in adolescence and psychological distress in adults: A cross-sectional study based on a retrospective recall
Source: PLoS One. 2019 Nov 21;14(11):e0225454. doi: 10.1371/journal.pone.0225454 (PMC6872135; doi:10.1371/journal.pone.0225454)
Supplement: S1 Table — (DOCX) [file pone.0225454.s001.docx]

**Table 1. Demographics and psychosocial characteristics of the participants (N=2,606)**

|  | N (%) | Mean (SD) |
| --- | --- | --- |
| **Age (mean)** |  | 38.08 (7.03) |
| **Sex (men)** | 1153 (44.2) |  |
| **Married** (yes) | 1962 (75.3) |  |
| **Education** |  |  |
| Junior high school | 79 (3.0) |  |
| High school | 514 (19.7) |  |
| Some college | 909 (34.9) |  |
| University or higher | 1104 (42.4) |  |
| **Employment** |  |  |
| Working | 2018 (77.5) |  |
| On leave | 61 (2.3) |  |
| Job seeking or House wife or Students | 527 (20.2) |  |
| **Household Income (per year)** |  |  |
| ≦2.5 million yen | 177 (6.8) |  |
| ≦5 million yen | 411 (15.8) |  |
| ≦7.5 million yen | 611 (23.4) |  |
| ＞7.5 million yen | 825 (31.7) |  |
| Not known | 582 (22.3) |  |
| **Current or ever smoking (y**es) | 1181 (45.3) |  |
| **Drinking alcohol** |  |  |
| More than 3 times per week | 881 (33.8) |  |
| Less than 3 times per week | 1725 (66.2) |  |
| **Living in 15 years old (Poor)** | 492 (18.9) |  |
| **K6: Psychological distress** |  | 4.09 (4.55) |
