# Supplementary material for: Personal values in adolescence and psychological distress in adults: A cross-sectional study based on a retrospective recall
Source: PLoS One. 2019 Nov 21;14(11):e0225454. doi: 10.1371/journal.pone.0225454 (PMC6872135; doi:10.1371/journal.pone.0225454)
Supplement: S2 Table — (DOCX) [file pone.0225454.s002.docx]

**Table 2.** **Personal Values in Adolescence and Psychological Distress in Adulthood: Multiple Linear Regression Analysis of Data from Community Residents in Japan (N=2,606)**

|  | | **Model 1** | | | **Model 2** | | | **Model 3** | | |
| --- | --- | --- | --- | --- | --- | --- | --- | --- | --- | --- |
|  |  | Ｂ | SE | *p* | Ｂ | SE | *p* | Ｂ | SE | *p* |
| **Areas of value priority: (1-7)** | Mean (SD) |  |  |  |  |  |  |  |  |  |
| **Avoiding causing trouble** | 5.58 (1.36) | 0.07 | 0.08 | 0.34 | 0.09 | 0.08 | 0.23 | 0.10 | 0.08 | 0.21 |
| **Positive evaluation** | 4.90 (1.40) | 0.09 | 0.07 | 0.24 | 0.08 | 0.07 | 0.25 | 0.09 | 0.07 | 0.24 |
| **Belief** | 4.83 (1.41) | 0.09 | 0.08 | 0.26 | 0.10 | 0.08 | 0.24 | 0.10 | 0.08 | 0.22 |
| **Financial success** | 4.22 (1.53) | 0.10 | 0.07 | 0.18 | 0.09 | 0.07 | 0.26 | 0.05 | 0.07 | 0.47 |
| **Improving society** | 3.77 (1.43) | 0.04 | 0.08 | 0.68 | 0.06 | 0.08 | 0.49 | 0.07 | 0.08 | 0.40 |
| **Pursuing one’s interest** | 5.13 (1.40) | 0.17 | 0.08 | 0.03* | 0.18 | 0.08 | 0.02* | 0.19 | 0.08 | 0.01* |
| **Social influence** | 3.31 (1.41) | -0.06 | 0.08 | 0.50 | -0.07 | 0.08 | 0.39 | -0.07 | 0.08 | 0.40 |
| **Enduring active challenging** | 4.50 (1.43) | -0.15 | 0.08 | 0.06 | -0.16 | 0.08 | 0.048* | -0.17 | 0.08 | 0.04* |
| **Cherishing family and friends** | 5.54 (1.25) | -0.37 | 0.08 | <0.01** | -0.39 | 0.08 | <0.01** | -0.38 | 0.08 | <0.01** |
| **Graduating from school** | 4.26 (1.67) | -0.03 | 0.07 | 0.71 | -0.01 | 0.07 | 0.85 | 0.004 | 0.07 | 0.95 |
| **Stable lifestyle** | 4.86 (1.43) | 0.04 | 0.08 | 0.66 | 0.05 | 0.08 | 0.51 | 0.05 | 0.08 | 0.55 |
| **Commitment to values (8-40)** | 26.34 (4.76) | -0.05 | 0.02 | 0.02* | -0.05 | 0.02 | 0.02* | -0.05 | 0.02 | 0.02* |
| **Intercept** | | 10.64 |  |  | 9.94 |  |  | 9.61 |  |  |
| **Adjusted R-square** | | 0.074 | | | 0.077 | | | 0.084 | | |

**§ Unstandardized regression coefficient (B) for one-point increase in the score is shown.**

**Sociodemographic variables were adjusted for in the Model 1; smoking and alcohol drinking were adjusted for in model 2; poor economic status at age 15 was adjusted for in model 3**

****p* < 0.05, ** *p* < 0.01**
